# Seasonal variation of stroke incidence in Wujin, a city in southeast China

**DOI:** 10.1002/hsr2.29

**Published:** 2018-03-09

**Authors:** Huafeng Jin, Zhiliang Xu, Yunyan Li, Jiaping Xu, Hongmei Shan, Xiaoli Feng, Yan Xie, Keyu Bian, Dong Qin

**Affiliations:** ^1^ Department of Neurology Wujing Traditional Chinese Medicine Hospital Changzhou Jiangsu Province China; ^2^ Chang Zhou Meteorological Bureau Chang Zhou Jiangsu Province China

**Keywords:** Chinese, incidence, seasonal variation, stroke

## Abstract

**Objective:**

This study aimed to evaluate seasonal variations in stroke incidence in a city in southeast China.

**Methods:**

First‐ever stroke in residents aged 20 or older in Wujin city was reported by local physicians between January 2006 and December 2007. All reported stroke cases were checked with the city‐wide health insurance system. Fatal cases were checked with the household registry system. The annualized stroke incidences were calculated for each month and each season. The stroke incidence was compared among seasons, with spring as a reference.

**Results:**

Wujin city had an adult (≥20 y old) population of 1 278 020 in 2010. A total of 2319 stroke cases, 1217 male and 1102 female, were reported in 2006 and 2007. The raw annual incidence of stroke was 90.7/100 000 (95% CI: 82.1‐94.5). Stroke incidence was highest in September (125.2/100 000; 95% CI = 109.4‐139.3) and lowest in January (78.8/100 000; 95% CI = 69.1‐93.2). Stroke incidence was highest in autumn (OR = 1.18, 95% CI: 1.05‐1.32, P < .001, compared with spring). Autumn also had the highest incidences of ischemic stroke (OR = 1.22, 95% CI: 1.06‐1.40).

**Conclusions:**

Stroke incidence showed monthly and seasonal variations in southeast China. Autumn had the highest incidences of both ischemic stroke and cerebral hemorrhage.

## 1 INTRODUCTION

Seasonal fluctuations of stroke incidence have been investigated in many studies, but with inconsistent results.^1^, ^2^, ^3^, ^4^, ^5^, ^6^, ^7^, ^8^ While some studies have reported increased stroke incidence, mortality, and hospitalization during the colder winter, and decreased ones during the warmer summer,^1^, ^2^, ^3^, ^4^, ^5^ others have reported the reverse^6^ or have failed to detect significant seasonal variations.^7^, ^8^ These inconsistencies may be attributed partly to differences in sample population, local meteorological profiles, and study methodology and thus warrant further study.

Previous studies indicated that local geographic and climatic profiles may be an important factor influencing seasonal variation in stroke incidence.^9^ For example, seasons tend to be more distinctive in high‐latitude than in low‐latitude regions. Circannual variations of temperature, rainfall, and humidity, all of which have been associated with stroke risk,^10^ tend to be stronger in continental than in oceanic climates.^11^ Seasonal variation of stroke incidence, therefore, may be more prominent in high‐latitude regions with continental climates. In this study, we examined the monthly and seasonal variation of stroke incidence in Wujin, a city located in southeast China, and with 4 distinctive seasons. The city has an adult population of more than 1 million, which allows such incidence evaluation.^12^

## 2 METHODS

### 2.1 Season and climate profiles in Wujin City

Wujin was classified a county in southeast China before 1995. Between 1995 and 2002, it was administrated as a city, and it then merged into Changzhou city. Since then, it has been administrated as a district. Traditionally and geographically, however, Wujin district can be regarded as a city, with a population of more than 1 million, and with a clear city boundary. The city is situated in the Yangtze River delta. It is located between Nanjing and Shanghai, the 2 largest cities in southeast China. Wujin City covers an area of 1266 square kilometers (northern latitude 31°19′‐31°55′, east longitude 119°38′‐120°12′). Climatologically, the area belongs to the north subtropical zone. The annual average temperature of the area, between 2002 and 2007, has been 17.28°C. The average temperature in spring, summer, autumn, and winter has been 16.9°C, 27.5°C, 18.1°C, and 4°C, respectively. In 2007, the highest temperature was 38.7°C, and the lowest temperature was −3.4°C. The average annual precipitation was 1117 mm, most of which took place in the summer (613 mm, 54.9%). Rainy season usually lasts from June to July. The average annual sunshine is 2085 hours. The weather in Wujin city follows 4 very distinct seasons: spring, summer, autumn, and winter. Because the city is 300 km away from the Pacific Ocean, typhoons are rare and mild.

### 2.2 Population characteristics of Wujin City

According to the results of the 2010 China National Population Census, about 85% of Wujin residents live in a rural area. The population of Wujin City has remained relatively stable over the past 30 years. The total population was 1 560 000 in 2010. The population of residents aged 20 or older was 1 278 020. As a city located in southeast China where socioeconomic development is relatively advanced, few inhabitants have emigrated to other cities in the last 30 years, but the city has received a large number of temporal immigrants from other parts of China, who usually work in Wujin for several years and then go back to their hometowns. According to local regulations, all permanent residents are covered with government‐supported health care insurance system. Immigrants usually have health insurance in their hometown. Therefore, these temporal residents were not included in the present study.

### 2.3 Stroke diagnosis and case confirmation

The enrolled patients included all residents aged 20 or older in Wujin City hospitalized with a first‐ever stroke in the city hospitals or clinics between January 1, 2006, and December 31, 2007. We excluded very young stroke cases (<20 years old) in this study because stroke in children and juvenile population is relatively rare, and etiologies of children stroke may be different from those in adult stroke.^13^ There were 17 hospitals and 329 physicians included in the study. All of these hospitals are qualified for managing acute stroke patients, and all physicians were certified neurologists. Stroke was diagnosed, classified, and reported by physicians in the local hospitals and clinics. Patients with transient ischemic attack (TIA) and without a subsequent stroke were not included. Stroke was defined as sudden onset of neurological symptoms, which continued for at least 24 hours unless interrupted by death or artery recanalization treatment (eg, thrombolysis or thrombectomy).^14^ After a patient was diagnosed with stroke, previous medical history was reviewed to exclude recurrent stroke. Stroke was categorized as cerebral infarction, intracerebral hemorrhage, and subarachnoid hemorrhage. Classification of stroke type and subtype was based on clinical symptoms and neurological imaging examinations such as CT or MRI. Presence of major cardiovascular risk factors, such as hypertension, diabetic mellitus (DM), and smoking, was determined according to patient reports, physical examinations, and laboratory tests. Date and time of stroke onset were retrospectively retrieved from medical records and charts. For wake‐up stroke, time of stroke onset was determined as the last known normal time.

Because each stroke patient can be reimbursed if he or she is a permanent resident of Wujin City, we could confirm stroke cases by the local health insurance system. With this strategy, we could exclude stroke patients who were not permanent residents of the city. By checking the reimbursement database, we could also include some neglected cases by further rechecking the medical records. Out‐of‐hospital deaths were checked by local vital registry system. This study was approved by the Institutional Review Boards of the local participating hospitals. Each hospitalized patient was invited to sign an informed consent for using that their data in future research.

### 2.4 Statistical methods

This study covered the period between January 1, 2006, and December 31, 2007. During this 24‐month period (730 days), the monthly and seasonal stroke incidences were calculated and compared. The year was divided into 4 seasons: winter included December, January, and February; spring included March, April, and May; summer included June, July, and August; and autumn included September, October, and November. To examine the effect of age on the patterns of stroke incidence, patients were stratified into groups younger than 65 and those 65 years or older at the time of stroke onset.

Annual incidence was defined as rates per 100 000 per year. Rate ratios were calculated with spring as reference. Annual incidence rates of stroke per 100 000, including 95% CI, were calculated for each month and each season. To calculate the annualized incidence of stroke in a given month, the total number of stroke cases within the month was divided by the number of subjects at risk within the city across the 2 years of available data. For example, the annualized stroke incidence for January was calculated by the case numbers in January divided by the population at risk, and then divided by number of involved years (2), and then further divided by proportion of day in January in a year (31/365). Annualized stroke incidences in seasons were calculated using the same method. The population demographic data were derived from 2010 China National Population Census.

Stroke incidences in different months were ranked and compared using the chi‐square test. The relative risk of stroke occurrence was evaluated with the month of lowest stroke incidence set as reference. Using the same methods, stroke incidences in seasons were evaluated and compared. The odds ratio (OR) was calculated separately by sex, age group, risk factor strata, and by stroke subtypes.

All statistical analyses were performed using SPSS for MS Windows, version 13.0 (SPSS Inc). An α level of 0.05 was deemed as statistically significant.

## 3 RESULTS

The total population of Wujin city was 1 560 000, and the population of residents aged 20 or older was 1 278 020 in 2010. There were 2319 stroke cases (male, 1217; female, 1102) during the 2‐year survey. The average age of stroke cases was 68.1 ± 12.3. The raw stroke incidence was 90.7/100 000 (95% CI = 82.1‐94.5) annually. The characteristics of stroke cases and that of the overall Wujin population aged 20 or older were compared, and the results are shown in Table 1. There were 1562 (67.4%) stroke patients aged 20 to 64 at time of stroke onset. Hypertension was present in 1633 (70.45%) of stroke patients. DM was present in 293 (10.3%) of the stroke patients. There was a history of smoking in 284 (12.2%) stroke cases. Compared with the general population,^11^, ^12^, ^13^ prevalences of hypertension, DM, and smoking were significantly higher in stroke cases (*P* < .01).

**Table 1:** Characteristics of stroke cases vs total Wujin population aged 20 or older

Characteristic	Stroke Cases, N = 2319	Wujin Population Aged 20 or Older, N = 1278020
Male, n (%)	1217 (52.5%)	658699 (51.5%)
Aged 20‐64 y, n (%)	1562 (67.4%)	1146224 (89.7%)
Aged 65 or older, n (%)	757 (32.6%)	131796 (10.3%)
Hypertension, n (%)	1633 (70.4%)	240268 (18.8%)^a^
Diabetes mellitus, n (%)	293 (10.3%)	33229 (2.6%)^b^
Smoking, n (%)	284 (12.2%)	437083 (34.2%)^c^

a Hypertension prevalence was retrieved from a national survey of a sample population >15 y old.^15^

b DM prevalence was retrieved from a national survey of a sample population 20‐74 y old.^16^

c Smoking prevalence was retrieved from a national survey of a sample population 15‐69 y old.^17^

**Figure 1:**
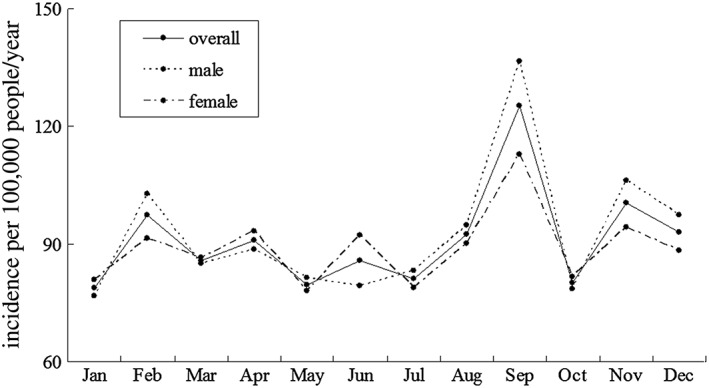
Monthly fluctuation of stroke incidence in male, female, and overall adult populations

**Figure 2:**
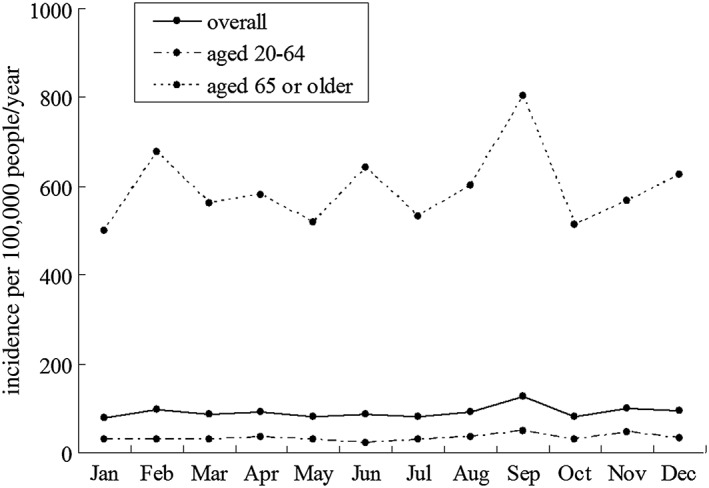
Monthly fluctuation of stroke incidence in populations aged 20‐64 and ≥65

**Figure 3:**
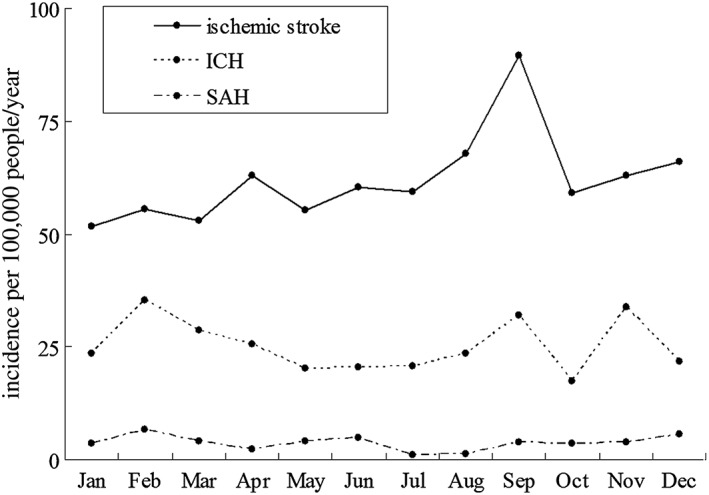
Monthly fluctuation of ischemic stroke, intracerebral hemorrhage, and subarachnoid hemorrhage incidences

Figure 1 shows the monthly fluctuation of stroke incidence in male, female, and overall adult population. There is 1 peak of monthly stroke incidence in male, female, and overall adult population. Annual stroke incidence per 100 000 was highest in September (125.2; 95% CI = 109.4‐139.3) and lowest in January (78.8; 95% CI = 69.1‐93.2). September had a 58.9% higher stroke incidence compared with January (RR = 0.650, 95% CI = 0.536‐0.788, *P* < .05). Stroke incidence in September was highest in both men (136.7; 95% CI = 114.8‐158.3) and women (113.0; 95% CI = 92.8‐133.7). Figure 2 shows the monthly fluctuation of stroke incidence in population aged 20 to 64, and population aged 65 or older. Stroke incidence in September was highest both in residents aged 20 to 64 years (47.2; 95% CI = 37.9‐57.3) and in those aged 65 years or older (803.1; 95% CI = 682.9‐918.8). Figure 3 shows the monthly fluctuation of different types of stroke: ischemic stroke, intracranial cerebral hemorrhage, and subarachnoid hemorrhage. Ischemic stroke had the highest incidence in September (89.5; 95% CI = 76.5‐101.8). The monthly fluctuation concerning incidences of cerebral hemorrhage and subarachnoid hemorrhage was not statistically significant.

Spring had the lowest stroke incidence. The relative stroke risk in each season was evaluated with stroke incidence with spring as reference (Table 2). Stroke incidence was highest in autumn (OR = 1.18, 95% CI: 1.05‐1.32, *P* < .001). Autumn also had the highest incidences of cerebral infarction (OR = 1.22, 95% CI: 1.06‐1.40). The seasonal fluctuation concerning incidences of cerebral hemorrhage and subarachnoid hemorrhage was not statistically significant.

**Table 2:** Relative risk of stroke by seasons, OR (95% CI) with spring as reference

	Spring	Summer	Autumn	Winter
All stroke	1	1.013 (0.900‐1.139)	1.178 (1.052‐1.320)	1.025 (0.912‐1.153)
Men	1	1.011(0.857‐1.192)	1.245 (1.064‐1.456)	1.060 (0.901‐1.248)
Women	1	1.015 (0.857‐1.201)	1.108 (0.940‐1.307)	0.989 (0.834‐1.172)
Aged 20‐64	1	0.901 (0.730‐1.113)	1.297 (1.069‐1.573)	0.962 (0.781‐1.183)
Aged 65 or older	1	1.068 (0.926‐1.232)	1.120 (0.973‐1.289)	1.057 (0.917‐1.219)
With hypertension	1	0.958 (0.834‐1.101)	1.089 (0.951‐1.246)	0.975 (0.848‐1.120)
Without hypertension	1	1.167 (0.934‐1.458)	1.431 (1.156‐1.770)	1.167 (0.934‐1.458)
With DM	1	1.028 (0.741‐1.426)	1.085 (0.785‐1.498)	1.014 (0.730‐1.408)
Without DM	1	1.010 (0.891‐1.147)	1.192 (1.056‐1.346)	1.027 (0.906‐1.165)
Smoking	1	0.900 (0.623‐1.300)	1.650 (1.197‐2.274)	1.183 (0.839‐1.669)
No smoking	1	1.027 (0.906‐1.163)	1.120 (0.992‐1.266)	1.006 (0.888‐1.140)
Cerebral infarction	1	1.098 (0.953‐1.265)	1.221 (1.063‐1.401)	0.992 (0.858‐1.147)
Cerebral hemorrhage	1	0.869 (0.692‐1.090)	1.100 (0.888‐1.363)	1.044 (0.840‐1.296)
Subarachnoid hemorrhage	1	0.652 (0.340‐1.250)	1.043 (0.589‐1.849)	1.435 (0.843‐2.443)

All are evaluated by Pearson test.

## 4 DISCUSSION

In this study, we found a significant monthly fluctuation of stroke incidences in a city located in southeast China. Both overall stroke incidence and ischemic stroke incidence were highest in September.

In most previous studies, the highest incidence of stroke was found in the colder winter or spring.^1^, ^2^, ^3^, ^4^, ^5^ A recent national study in Finland reported that all major stroke subtypes occurred most commonly in autumn and less frequently in summer.^18^ In this study, we found the incidence of stroke to be highest in September (autumn). These disagreements may reflect local seasonal variations and demographic profiles of the study population. In Wujin City, July and August have the hottest and wettest days of the year, while December and January have the coldest and driest days in the year. Weather changes gradually from hot to cold and from wet to dry in September and October. Blood pressure and blood glucose levels may change to adapt to this changing environment.^19^ The weather changes may have resulted in fluctuation of these physical parameters and subsequently influenced the risk of cardiovascular diseases.^19^, ^20^ This hypothesis has been supported by a recent study, in which a positive association between diurnal temperature range and risk of cardiovascular diseases was established.^21^

Why stroke risk varies among seasons is not fully understood. Change of physiological processes adapted to the weather could serve as a trigger for stroke occurrence.^22^ Variations in temperature among seasons have been presumed to be a possible cause underlying seasonal variation of stroke risk.^23^ Cold and dry weather could result in increased blood pressure, hematological changes, and respiratory infections.^24^ The cold and dry weather may enhance evaporation load and increase physical need for salt and subsequently increased blood pressure and stroke risk. The cold weather may also limit outdoor physical activity and enhance fat reservation^25^, ^26^ and consequently increase risk of obesity and related metabolic abnormalities.^27^, ^28^ In the cold weather, residents tend to consume more alcohol, which may increase alcohol‐related stroke risk.^29^

Seasonal pattern of influenza epidemic, air pollution, and other respiratory tract infections are also presumed to influence the seasonal variation of stroke risk. An increasing number of studies have linked ambient air pollution with the risk of ischemic stroke.^30^ The potential effective pollutants include SO_2_, NO_2_, O_3_, NO, CO, and fine particulate matter (PM <2.5 μm in diameter, PM2.5).^31^, ^32^ The pollutants may be diluted in the rainy days. As in this study, Wujin city has the most rainfall in the summer, so air pollution grade is lowest in the summer. The fluctuation of air pollution among seasons could also influence stroke risk.^30^ This study indicated that the rise of ischemic stroke incidence in autumn is more prominent in men and in smokers. Men usually stay longer time in the open air. They are more likely to be influenced by air pollution and dramatic weather changes, both of which may increase the risk of ischemic stroke. Smoking and air pollution may synergistically cause hazardous effects, but this possibility has not been investigated to date.

Several limitations of this study should be emphasized when interpreting the results. Stroke cases were diagnosed by local doctors from 17 hospitals and 329 clinics without unified training. Although they used the same criteria for stroke diagnosing, the clinical protocol and neurological procedures may have been different. This may have resulted in discrepancies in stroke diagnosis and classification among hospitals and among doctors. All stroke patients were screened from hospital records. This strategy may underestimate the stroke incidence, because severe patients who died before hospitalization, and patients with mild stroke may have been missed, although some fatal stroke patients were enrolled by household registry system. This study covered only 2 years, which cannot exclude variations of stroke risk among years. Months were included in 4 seasons arbitrarily, but it may be more reasonable to divide some months into 2 parts and included them in 2 seasons. The study was based on data from 10 years ago, and the current stroke incidences in this area may have changed to some extent. The results may not be generalizable to other populations.

In conclusion, we found increased stroke incidence in September (autumn) in a city of southeast China. This increased risk of stroke was observed in both young and old subjects and in both men and women. There was increased incidence of intracerebral hemorrhage and subarachnoid hemorrhage in February. This monthly fluctuation of stroke incidences may be attributed to the transitional changes of weather in September and February in this city. These results may have important implications for preventing stroke in the population.

## CONFLICT OF INTEREST

None declared.

## AUTHOR CONTRIBUTION

Conceptualization: Huafeng Jin, Zhiliang Xu

Formal Analysis: Keyu Bian, Dong Qin

Funding Acquisition: Huafeng Jin, Zhiliang Xu

Writing ‐ original draft: Huafeng Jin

Writing – review and editing: Yunyan Li, Jiaping Xu, Hongmei Shan, Xiaoli Feng, Yan Xie, Keyu Bian, Dong Qin
